# Effect of Glucocorticoid Use in Patients With Biopsy-Proven Acute Interstitial Nephritis: Insights From a Colombian Cohort

**DOI:** 10.1155/ijne/9980649

**Published:** 2025-04-18

**Authors:** Joaquín Rodelo-Ceballos, Camilo García-Prada, Mauricio Restrepo-Escobar, Laura Lopera-Restrepo, Angie Pinto-Diaz, Luis Fernando Arias-Restrepo

**Affiliations:** ^1^Section of Nephrology, Department of Internal Medicine, Universidad de Antioquia, Medellín, Colombia; ^2^Section of Nephrology, Department of Internal Medicine, Hospital Universitario San Vicente Fundación, Medellín, Colombia; ^3^Section of Rheumatology, Department of Internal Medicine, Universidad de Antioquia, Medellín, Colombia; ^4^Department of Pathology, Universidad de Antioquia, Medellín, Colombia

**Keywords:** acute interstitial nephritis (AIN), acute kidney injury (AKI), clinical research, cohort study, glucocorticoids (GCs), kidney replacement therapy (KRT)

## Abstract

**Introduction:** Acute interstitial nephritis (AIN) is a major cause of acute kidney injury, commonly triggered by medications or infections. Although glucocorticoid (GC) therapy is recommended for patients who do not improve after removing the suspected cause, the evidence supporting its use remains limited.

**Materials and Methods:** This retrospective cohort study was conducted at the Pathology Unit of the University of Antioquia—San Vicente Fundación Hospital in Medellín, Colombia, reviewing patients aged 14 and older with biopsy-proven AIN over an 11-year period. Two groups were formed based on whether or not they received GC treatment. Key outcomes included changes in delta creatinine (serum creatinine change from peak to 6-month follow-up) and the need for permanent kidney replacement therapy. Linear regression analyses assessed factors influencing delta creatinine at 6 months, adjusting for age, clinical severity, time to GC initiation, and histological findings.

**Results:** Of 139 eligible patients, 101 received GC therapy. The GC-treated group showed a significantly greater reduction in delta creatinine compared to the nontreated group (−2.3 mg/dL; 95% CI, −3.6 to −1.1, *p* < 0.001). Multivariate analysis identified GC therapy as an independent predictor of improved kidney function (delta creatinine reduction: −1.47 mg/dL; 95% CI, −2.68 to −0.27, *p*=0.017), particularly when initiated within 7 days of diagnosis. The GC-treated group also had a lower incidence of permanent dialysis dependence (54% at admission vs. 11% at 6 months). Adverse events occurred in 20.1% of the cohort, with a higher frequency in the GC group (*p*=0.076).

**Conclusion:** GC therapy may improve kidney outcomes in patients with biopsy-proven AIN, especially when initiated early. These results support the need for prospective studies to further evaluate its efficacy in AIN management.

## 1. Introduction

Acute interstitial nephritis (AIN) is a common cause of acute kidney injury (AKI). It is characterized by inflammation of the kidney interstitium, which may be accompanied to a varying extent by tubular, interstitial, vascular, and glomerular involvement. Despite being a histopathological diagnosis, not all patients undergo kidney biopsy. The incidence of AIN in hospitalized patients ranges from 5% to 27%, representing the third leading cause of AKI in this population [1, 2].

The etiology of AIN is diverse and includes drug-induced cases (60%–70%), those secondary to infections, those associated with systemic diseases, and those related to herbal products. Seventy percent of AIN cases are attributed to drug-induced allergic reactions, with antibiotics, proton pump inhibitors, and nonsteroidal anti-inflammatory drugs (NSAIDs) being the most common culprits. These medications are widely used in the general population and are available over the counter in many countries [3, 4].

The clinical features of AIN are nonspecific and may include general discomfort, nausea, and vomiting. Hypersensitivity reactions in drug-induced cases may manifest with fever, arthralgia, skin rash, and eosinophilia; however, these symptoms occur in only 10% of patients [5, 6]. Usually, the reaction to medications occurs within the first few days of exposure; however, the onset of drug-induced acute interstitial nephritis (DI-AIN) may be delayed by weeks or months after drug initiation, posing challenges in diagnosis [6].

Urine dipstick testing may reveal hematuria, sterile pyuria, and low-grade proteinuria, but these characteristics may be absent. Given the lack of reliable clinical signs, a kidney biopsy is required for diagnosis [5, 7]. Biopsy typically shows an inflammatory infiltrate of lymphocytes, plasma cells, and macrophages, with tubulitis, tubular degeneration, and sometimes noncaseating granulomas. As AIN progresses, interstitial fibrosis and tubular atrophy (IF/TA) may lead to kidney failure, requiring long-term kidney replacement therapy (KRT). AIN is a treatable cause of AKI if detected early and addressed promptly and yet remains the leading cause of kidney failure in 3%-4% of incident patients [5, 7–10].

Treatment typically involves discontinuing the causative agent and optimizing kidney function. Glucocorticoid (GC) therapy is commonly used in patients who do not show improvement. However, the efficacy of GC in treating AIN remains uncertain due to the absence of controlled trials and inconsistent findings in existing studies [1, 10–12]. This research seeks to evaluate the impact of GC therapy on clinical outcomes in biopsy-confirmed AIN patients within a Colombian cohort.

## 2. Materials and Methods

### 2.1. Design, Setting, and Participants

This retrospective cohort study was conducted at the Pathology Unit of the University of Antioquia—San Vicente Fundación Hospital (HSVF) in Medellín, Colombia. HSVF serves as a referral center for kidney diseases and nephropathology, covering a population of over 8 million people in the northeast region of the country [13]. Kidney biopsies diagnosed with AIN at HSVF between August 2011 and December 2021 were reviewed. Given that this period spanned the COVID-19 pandemic, it is important to note that no patients with COVID-19 or receiving treatment for COVID-19-related conditions were included in the study cohort.

Patients aged 14 years and older with biopsy-confirmed AIN, due to either unexplained AKI or medication-induced AIN, were eligible for inclusion. Diagnostic criteria for AIN included the presence of a diffuse inflammatory cellular infiltrate surrounding nonatrophic tubules, with tubulitis and varying degrees of interstitial edema and fibrosis, in the absence of bacterial infection [1, 5–10, 14, 15]. Exclusion criteria included IgG4-related disease, sarcoidosis, coexisting glomerulonephritis, follow-up of less than 3 months, or insufficient clinical information. Additionally, patients receiving maintenance GCs for other diagnoses were excluded.

### 2.2. Variables

The primary exposure was the administration of GC, as determined by the treating physician. The standard protocol at HSVF included an initial intravenous (IV) bolus of methylprednisolone at a dose of 250 mg daily for 3 days. This was followed by oral prednisolone at 0.7 mg/kg/day for 2 weeks, tapered over 4–6 weeks. For patients who did not receive IV methylprednisolone, an alternative regimen of oral prednisolone at 1 mg/kg/day for 2 weeks was administered, tapered over 4–6 weeks. The primary outcome was the change in serum creatinine (delta creatinine), defined as the difference between serum creatinine at 6-month follow-up and the peak creatinine during acute AIN. For patients requiring dialysis at biopsy, the peak creatinine before dialysis initiation was recorded. Secondary outcomes included KRT for more than 3 months, indicative of end-stage kidney disease (ESKD).

A sensitivity analysis was conducted using the change in creatinine from peak levels to discharge, allowing for the inclusion of 161 patients instead of 139. This adjustment accounted for 22 patients who lacked a creatinine measurement at the 6-month outpatient follow-up but had a serum creatinine value on the day of hospital discharge. The analysis aimed to mitigate potential biases from missing follow-up data and provide a more comprehensive assessment of treatment effects.

### 2.3. Data Sources and Measurement Methods

All patients were hospitalized during DI-AIN and discharged once their kidney function showed signs of improvement or stabilization. If the causative drug was identified, it was discontinued in all cases. Following discharge, most patients were followed up at regular intervals until complete kidney recovery or for a minimum of 6 months. Data were extracted from clinical records, including demographic information, clinical findings, laboratory results (e.g., differential leukocyte count, biochemical profile, urine sediment, and 24 h proteinuria), and estimated glomerular filtration rate (eGFR), calculated using the CKD-EPI 2009 formula for adults only. The responsible drug for AIN and the time between drug withdrawal and GC initiation were also recorded. Additionally, the highest serum creatinine value, the need for acute KRT, and whether the patient remained on dialysis for more than 3 months were documented. Serum creatinine at discharge was defined as the value recorded just before hospital discharge, while final creatinine was defined as the value obtained at 6 months of follow-up.

All biopsies were processed by the Department of Pathology at the University of Antioquia and reviewed by an expert nephropathologist [13, 15]. Kidney biopsy samples contained at least 10 glomeruli for evaluation, and both light microscopy and immunofluorescence were performed for diagnosis. Acute tubulointerstitial lesions were defined as interstitial inflammation without IF, while chronic lesions were characterized by IF/TA. These lesions were assessed using hematoxylin and eosin, periodic acid–Schiff, Masson's trichrome, and silver methenamine stains. AIN severity was graded based on the percentage of interstitial inflammation, while IF was categorized as absent, mild (< 25%), moderate (26%–50%), or severe (> 50%). The percentage of glomeruli with global sclerosis was also recorded.

### 2.4. Statistical Methods

Categorical variables were expressed as frequencies and percentages, while continuous variables were presented as means and standard deviations or medians and interquartile ranges, depending on the normality of the data (assessed using graphical methods and the Shapiro–Wilk test). Univariate and multivariate analyses were performed using multiple linear regression. Analyses were adjusted for patient age, the need for acute KRT, and the severity of the clinical condition to account for potential confounding factors, such as differing kidney reserve, greater degree of atrophy, or treatment indications. A *p* value of less than 0.05 was considered statistically significant. Statistical analysis was performed using STATA 16.0.

## 3. Results

A total of 175 patients with AIN were identified, with 44 excluded—14 due to insufficient clinical information, including those referred from other hospitals solely for kidney biopsy without follow-up, and 22 due to a follow-up of less than three months, often because they lived in rural areas or did not return for consultations. Consequently, 139 patients were included in the primary outcome analysis, and the majority of these patients (93%) self-identified as Latino or Hispanic. The median age at presentation was 45 years (IQR 28–62), and 56% of the patients were male. The median hospital stay was 12 days (IQR 9–21), and the median follow-up duration was 10 months (IQR 3–16). The most common etiology was antibiotic use, identified in 76 patients (54.6%), followed by NSAIDs in 37 cases (26.6%) and antivirals in 23 cases (16.5%). Only three patients (2%) exhibited the classic triad of rash, fever, and eosinophilia. Additionally, three cases were of unidentified origin. Further clinical and laboratory characteristics are presented in Table 1.

One hundred and one patients were treated with GC, while 38 were managed conservatively. All patients receiving GC were treated with oral prednisolone, and 61% initially received methylprednisolone boluses. Most patients received between 40 and 60 mg of oral prednisolone per day, while those receiving methylprednisolone were administered doses ranging from 250 to 500 mg for 3 consecutive days. Twenty-five patients (24.7%) were still on GC at 3 months with a median dose of 7.5 mg/day of prednisone, and the median treatment duration was 4 months (IQR 2 weeks to 6 months).

Baseline characteristics of the two groups based on treatment are shown in Table 1. Patients in the GC-treated group were older (50 years, IQR 31–64, vs. 37 years, IQR 26–49, *p*=0.008), had greater severity in clinical presentation (peak creatinine of 5.96 mg/dL, IQR 4.05–8.6, vs. 2.37 mg/dL, IQR 1.90–4.24, *p* < 0.001), and had a higher need of acute KRT (*n* = 55, 54% vs. *n* = 5, 13%, *p* < 0.001).

In the initial univariate analysis, the GC-treated group showed a greater reduction in delta creatinine (−2.3 mg/dL; 95% CI, −3.6–−1.1, *p* < 0.001). Upon adjusting for age, severity of clinical presentation, time from withdrawal of the offending drug and onset of GC, and the degree of IF/TA, the use of GC was associated with a significant reduction in delta creatinine (−1.47 mg/dL; 95% CI, −2.68 to −0.27, *p*=0.017). We also showed that the delta creatinine was greater if steroids were started early, especially before 7 days (−3.9 mg/dL; 95% CI, −7.4–−2.6) (see Table 2). No differences were observed when comparing NSAID use as a probable etiology. Additionally, in the GC-treated group, 44 out of 55 patients discontinued dialysis due to recovery of kidney function.

Adverse events were reported in 20.1% of the cohort, with a higher incidence in the GC-treated group (*p*=0.076). As expected, GC use increased the risk of infections, with 28 patients (20.1%) developing infections, including 6 cases of sepsis. Notably, infections were more prevalent among diabetic patients in the GC group. Of the 28 infected patients, 24 had comorbidities, including 19 with diabetes. While infections were more frequent in the GC group (23.7%) compared to the standard treatment group (10.5%), no deaths occurred due to infection (see Table 3).

### 3.1. Sensitivity Analysis

A sensitivity analysis was performed on 161 patients (114 treated with GCs and 47 managed conservatively). This analysis included 22 patients who lacked a creatinine measurement at the 6-month outpatient follow-up but had a serum creatinine value before hospital discharge, allowing for the calculation of the delta from peak. In this expanded analysis, only 14 of the initial 175 patients were excluded due to insufficient clinical information, as they were referred from other hospitals solely for kidney biopsy. The baseline characteristics of the sensitivity cohort were similar to those of the main cohort. See Supporting Table S1. Notably, this analysis was based on delta creatinine from peak levels to discharge from the hospital, rather than the 6-month follow-up, allowing us to assess the early effects of GC treatment on kidney function.

The sensitivity analysis demonstrated that GC treatment was associated with a greater reduction in delta creatinine compared to conservative management: −3.12 mg/dL (95% CI, −6.00 to −1.34) in the GC group versus −0.72 mg/dL (95% CI, −2.54 to −0.10) in the conservative group (*p* < 0.001). These results suggest that GCs have a significant early impact on kidney function recovery. Upon adjusting for age, severity of clinical presentation, time from withdrawal of the offending drug and onset of GC, and the degree of IF/TA, the use of GC was associated with a significant reduction in delta creatinine (−1.16 mg/dL; 95% CI, −1.92 to −0.20, *p*=0.015). For further details, see Supporting Table S2.

## 4. Discussion

In our cohort, primarily consisting of Latino or Hispanic patients with biopsy-proven AIN, a reduced GC regimen was associated with significant clinical benefits, both in the short term and at 6 months. This approach resulted in a notable reduction in delta creatinine levels at both hospital discharge and 6 months. The benefits were most pronounced in patients who started GC treatment within 7 days of diagnosis. Importantly, these positive outcomes were achieved with a manageable incidence of adverse events related to GC therapy.

The management of AIN with GCs remains controversial, as evidenced by the lack of evidence-based clinical practice guidelines. Various regimens exist, some incorporating methylprednisolone boluses and others not. Many centers follow a regimen of 500–1000 mg IV methylprednisolone daily for 3 days, followed by oral prednisolone at 1 mg/kg/day for two weeks, tapering over 8 weeks [5–7, 12, 14]. However, this approach may carry a high risk of adverse events. Our study clarifies that our modified GC regimen incorporating methylprednisolone boluses followed by a gradual 8-week oral prednisolone taper can effectively improve kidney outcomes while minimizing the risk of adverse events such as infections.

The favorable outcomes observed in the GC group were noteworthy despite the retrospective design, which introduces inherent biases and that patients who received GC tended to be older, with a typically greater compromise in eGFR, higher requirement for dialysis, and longer duration of AKI compared to those who solely discontinued the culprit medication. Our findings contribute to existing retrospective evidence indicating a potential beneficial effect of GC therapy on kidney function in patients with D-AIN [12, 16–18]. They also support the standardization of GC use in AIN patients admitted requiring dialysis or those who, despite discontinuing the offending agent for several days, still exhibit significant kidney function impairments. GC therapy should be initiated early after establishing the diagnosis, preferably within 1-2 weeks [6, 14, 16]. However, robust, high-quality evidence supporting this recommendation is currently unavailable.

Several retrospective studies have attempted to examine the effectiveness of GC in AIN patients with conflicting results (see Table 4). There remains a significant knowledge gap in the optimal use of GCs in AIN, as evidenced by varying treatment regimens across studies. For example, studies by González et al. [16] and Prendecki et al. [17] reported similar favorable outcomes with GC use, albeit with different regimens, highlighting the complexity of optimizing GC therapy in AIN. González et al. [16] in their logistic regression analysis showed that an interval longer than 7 days between drug withdrawal and the onset of steroid treatment, as well as the severity of IF, was the only clinical factors that significantly increased the risk of incomplete recovery of kidney function. In the UK cohort [17], after matching for some confounding factors, GC-treated patients showed higher eGFRs at 6 and 24 months, along with a potentially improved survival rate at 3 years, a finding not previously reported in other cohorts. Similar to Prendecki et al. [17], we also observed an improvement in eGFR from the time of biopsy to 6 months in the GC group, with a gain value of 24.5 mL/min compared to 16 mL/min reported by them.

On the other hand, studies like those by Clarkson et al. [11] and Muriithi et al. [1] did not demonstrate a beneficial effect of GC on AIN. Clarkson et al. [11] found no disparity in creatinine levels between GC-treated and untreated patients after 1 year. However, unlike Clarkson's study, our regimen extends beyond a 6-week timeframe, which could explain our favorable outcomes. Moreover, the relatively small sample size (60 patients) in Clarkson's study might have limited their ability to detect significant differences. Muriithi et al. [1] and Valluri et al. [19] also did not show a beneficial effect of GC, highlighting a worse prognosis associated with delayed initiation of steroids. In contrast, the majority of patients in our study commenced treatment within 7 days, with very few displaying IF/TA in biopsy samples.

In the study by Fernandez-Juarez et al. [12], 182 patients with biopsy-proven drug-induced AIN, approximately 41% of participants recovered and more than 75% of lost kidney function at 6 months, while 13% recovered less than 25%. Patients receiving GC within 15 days of diagnosis were more likely to achieve over 75% recovery than those recovering less than 25%. On average, patients received 13 weeks of GC therapy, with no association between the duration of therapy and kidney function recovery in univariate or multivariate analyses. Our study also demonstrated a favorable impact in terms of delta creatinine with GC even when initiated within 14 days of withdrawing the culprit drug. Regarding the duration of therapy, we tended to extend GC treatment to 16 weeks with a lower dose regimen, showing an acceptable rate of infections. Unfortunately, previous studies on GCs in AIN have not extensively reported adverse events [1, 12, 16, 17]. Nearly 20% of patients who received GC in our study experienced an episode of infection, with the majority being nonsevere, consistent with findings from reduced GC regimens in other contexts [20].

Our investigation contributes by proposing a standardized, reduced GC protocol that could guide clinical practice in similar settings. Comparing our results with other retrospective studies emphasized the need for standardized protocols validated through prospective trials.

Despite these strengths, our study has several limitations. As a retrospective series, it is prone to missing data and may introduce biases and deficiencies in the measurement and recording of some variables. The standardization of exposure variables was inconsistent in some cases, and their nonrandomized assignment complicates the interpretation of the relationship between exposure and outcome due to potential confounding factors. Although we attempted to control for confounders such as age, severity of clinical presentation, and the degree of IF/TA, some degree of residual confounding cannot be completely ruled out. Additionally, kidney function was assessed only at two time points—discharge and 6 months. While this approach offers an advantage over studies that rely solely on discharge creatinine, the relatively short follow-up limits our ability to evaluate long-term kidney function outcomes. Finally, the single-center design restricts the generalizability of our findings.

## 5. Conclusion

In conclusion, our study provides valuable insights into the management of AIN, advocating for the use of a reduced GC regimen to improve kidney outcomes. While our findings are promising, further prospective randomized controlled trials are needed to validate the efficacy and safety of GCs in diverse AIN patient populations. Addressing these knowledge gaps will be crucial for developing evidence-based guidelines for the optimal treatment of AIN, ultimately improving patient outcomes and reducing the burden of chronic kidney disease.

## Figures and Tables

**Table 1:** Clinical and laboratory characteristics of patients with acute interstitial nephritis.

Variable	Total *n* = 139	Treated with glucocorticoids (GCs) *n* = 101 (73%)	Standard treatment *n* = 38 (27%)	*p*
Male sex (%)	78 (56)	56 (55)	22 (58)	0.795

Age (years)	45 (28–62)	50 (31–64)	37 (26–49)	0.008

Latino or Hispanic (%)	129 (93)	92 (91)	37 (97)	0.202

*Comorbidities*				
None	63 (45.3%)	42 (41.5%)	21 (55.2%)	0.016
Hypertension	33 (23.7%)	24 (23.7%)	9 (23.6%)	
Diabetes	3 (2.1%)	2 (1.9%)	1 (2.6%)	
Hypertension and diabetes	26 (18.7%)	24 (23.7%)	2 (5.2%)	
HIV	6 (4.3%)	2 (1.9%)	4 (10.5%)	

Rash (%)	13 (9)	8 (8)	5 (13)	0.364

Fever (%)	36 (26)	24 (24)	12 (32)	0.382

Eosinophils (%)	190 (20–400)	200 (20–400)	115 (40–300)	0.528

Classic triad (%)	3	3	0	0.562

Proteinuria (mg/24 h)	482 (154–1000)	444 (150–916)	680 (290–1095)	0.211

Leukocyturia (HPF)	6 (1–20)	8 (2–25)	4 (1–10)	0.045

Hematuria (%)	76 (58)	62 (64)	14 (40)	0.014

Creatinine at admission (mg/dL)	3.7 (1.7–6.5)	4.5 (2.0–6.7)	1.8 (1.0–3.4)	< 0.001

eGFR at admission (mL/min) (‡): missing (8)	17.3 (6.9–44.8)	12.9 (6.35–38.9)	39.3 (21.0–77.1)	< 0.001

Peak creatinine (mg/dL)	5.1 (2.9–7.8)	5.96 (4.1–8.6)	2.37 (1.9–4.2)	< 0.001

*Fibrosis*				
< 25%	131 (94%)	94 (93%)	37 (97%)	0.148
26%–50%	6 (4%)	6 (6%)	0 (0%)	
51%–75%	1 (1%)	1 (1%)		
> 76%	1 (1%)	0 (0%)	1 (2%)	

*Tubular atrophy*				
< 25%	131 (94%)	94 (93%)	37 (97%)	0.148
26%–50%	6 (4%)	6 (6%)	0 (0%)	
51%–75%	1 (1%)	1 (1%)		
> 76%	1 (1%)	0 (0%)	1 (3%)	

Glomerulosclerosis	0 (0–0.14)	0 (0–0.2)	0 (0–0.07)	0.489

Need for acute KRT (%)	60 (43)	55 (54)	5 (13)	< 0.001

Duration on dialysis (days)	18 (7–30)	12 (7–21)	21 (6–75)^∗^	0.454

Interval between drug withdrawal and kidney biopsy (days)	7 (5–10)	7 (5–10)	7 (5–8)	0.029

Interval between diagnosis of AIN and GC (days)		1 (0.7–1.1)		N/A

*Note:* (‡):–eGFR not calculated for 8 patients under 18 years old (CKD-EPI formula not applicable). Values are presented as median (interquartile range) or *n* (%), unless otherwise specified.

Abbreviation: N/A, not available.

^∗^Four patients required acute KRT in the standard group. One received a single day of therapy, while another remained on KRT for 120 days before the biopsy during a prolonged hospitalization and continued KRT afterward.

**Table 2:** Kidney outcomes.

Variable	Total *n* = 139	Treated with glucocorticoids *n* = 101 (73%)	Standard treatment *n* = 38 (27%)	*p*
Creatinine at admission (mg/dL)	3.7 (1.7–6.5)	4.5 (2.0–6.7)	1.8 (1.0–3.4)	< 0.001
eGFR at admission (mL/min) (‡): missing (8)	17.3 (6.9–44.8)	12.9 (6.35–38.9)	39.3 (21.0–77.1)	< 0.001
Peak creatinine (mg/dL)	5.1 (2.9–7.8)	5.96 (4.1–8.6)	2.37 (1.9–4.2)	< 0.001
Creatinine at 6 months (mg/dL)	1.81 (1.1–2.6)	1.83 (1.20–2.9)	1.6 (1.0–2.1)	0.020
eGFR at 6 months (mL/min) (‡): missing (8)	42.2 (25.0–62.8)	37.3 (19.0–50.9)	54.5 (37.1–73.4)	0.005
Delta creatinine (mg/dL)	−2.9 (-5.4–−0.7)	−3.2 (−6.2–−1.4)	−0.8 (−3.0–−0.2)	< 0.001
Delta creatinine when interval between drug withdrawal and onset of GC treatment of ≤ 7 days (mg/dL)		−3.96 (−7.4–−2.6)	−0.8 (−3.0–−0.2)	< 0.001
Delta creatinine when interval between drug withdrawal and onset of GC treatment of > 7 days (mg/dL)		−2.75 (−6.12–−0.94)	−0.8 (−3.0–−0.2)	0.01
Delta creatinine when interval between drug withdrawal and onset of GC treatment of ≤ 14 days (mg/dL)		−3.63 (−6.26–−1.84)	−0.8 (−3.0–−0.2)	< 0.001
Delta creatinine when interval between drug withdrawal and onset of GC treatment of > 14 days (mg/dL)		−2.91 (−6.0–−0.94)	−0.8 (−3.0–−0.2)	0.09
Need for acute KRT (%)	60 (43)	55 (54)	5 (13)	< 0.001
Permanent dialysis (%)	12 (8.6)	11 (10.9)	1 (3)	0.734

*Note:* (‡):–eGFR not calculated for 8 patients under 18 years old (CKD-EPI formula not applicable). Values are presented as median (interquartile range) or *n* (%), unless otherwise specified.

Abbreviation: N/A, not available.

**Table 3:** Adverse events.

Variable	Total *n* = 139	Treated with glucocorticoids *n* = 101 (73%)	Standard treatment *n* = 38 (27%)	*p*
Infection				
No infection	106 (76.2%)	73 (72.2%)	33 (86.8%)	0.076
Any infection after GC or kidney biopsy	28 (20.1%)^∗^	24 (23.7%)	4 (10.5%)	
Type of infection				0.057
Urinary infection	12	11	1	
Respiratory infection	6	6	0	
Sepsis	6	5	1	
Skin infection	2	2	0	
Other	2	0	2	

^∗^Out of the 28 infected individuals, 24 have comorbidities, with 19 of them being diabetics. *n* (%).

**Table 4:** Studies on the use of glucocorticoids in acute drug-induced interstitial nephritis.

Author/year	Total *n* (GC/non-GC)	Peak-Cr (mg/dL) (GC)	Last-Cr (mg/dL) (GC)	Peak-Cr (mg/dL) (non-GC)	Last-Cr (mg/dL) (non-GC)	KRT at presentation (GC/non-GC)	KRT at discharge (GC/non-GC)
Clarkson et al. 2004	42 (26/16)	7.9	1.6	6.2	1.6	58% overall population/NA	6%/NA
Muriithi et al. 2014	95 (83/12)	4.5	1.4	3.0	1.5	24%/NA	4%/NA
Valluri et al. 2015	124 (73/51)	4.0	NA	3.2	NA	25%/16%	10%/10%
Gonzalez et al. 2008	61 (52/9)	5.9	2.1	4.9	3.7	NA	4%/44%
Prendecki et al. 2017	48 (45/3)	Aprox 3.7 (eGFR 17 mL/min)	Aprox 1.6 (eGFR 47 mL/min)	Aprox 1.6 (eGFR 38 mL/min)	Aprox 1.5 (eGFR 42 mL/min)	14%/0%	2%/0
Fernandez-Juarez et al. 2018	182 all on GC	5.7	Aprox 2.0 (eGFR 34 mL/min)	NA	NA	19%/NA	6%/NA
Raza et al. 2012	49 (37/12)	7.3	2.8	6.4	3.4	16%/42%	NA
Rodelo et al. 2023	139 (101/38)	5.9	1.8	2.4	1.6	54%/13%	11%/3%

*Note:* Cr, creatinine in mg/dL.

Abbreviations: eGFR, estimated glomerular filtration rate; GC, glucocorticoids; KRT, kidney replacement therapy; NA, not available.

## Data Availability

The data that support the findings of this study are available on request from the corresponding author. The data are not publicly available due to privacy or ethical restrictions.

## Supporting Information

Table S1. Clinical and laboratory characteristics of patients with acute interstitial nephritis according to glucocorticoid use or not.

Table S2. Renal outcomes of cohort patients according to glucocorticoid use or not.
