# Supplementary material for: Effect of Glucocorticoid Use in Patients With Biopsy-Proven Acute Interstitial Nephritis: Insights From a Colombian Cohort
Source: Int J Nephrol. 2025 Apr 18;2025:9980649. doi: 10.1155/ijne/9980649 (PMC12031597; doi:10.1155/ijne/9980649)
Supplement: Supporting Information — Additional supporting information can be found online in the Supporting Information section. [file 9980649.f1.docx]

**Supplementary tables**

Table S1. Clinical and Laboratory Characteristics of Patients with Acute Interstitial Nephritis

| Variable | Total  n = 161 | Treated with glucocorticoids (GC)  n = 114(71%) | Standard treatment  n = 47(29%) | p |
| --- | --- | --- | --- | --- |
|  | | | | |
| Male sex (%) | 90 (56%) | 63 (55%) | 27 (57%) | 0.800 |
| Age (years) | 39 (23-59) | 44.5 (25-63) | 33 (17-48) | 0.004 |
| Latino or Hispanic (%) | 150 (93%) | 104 (91%) | 46 (98%) | 0.178 |
| Rash (%) | 16 (10%) | 11 (10%) | 5 (11%) | 0.999 |
| Fever (%) | 45 (29%) | 31 (27%) | 14 (30%) | 0.752 |
| Eosinophils (%) | 200 (30 – 400) | 200 (20 – 400) | 100 (40 – 300) | 0.242 |
| Proteinuria (mg/24h) | 455 (166 – 1000) | 426 (150 – 916) | 585 (290 – 1040) | 0.139 |
| Leukocyturia (HPF) | 6 (2 – 20) | 8 (3 – 20) | 4 (1 – 16) | 0.052 |
| Hematuria (%) | 92 (60%) | 70 (61%) | 22 (47%) | 0.119 |
| Creatinine at admission (mg/dl) | 3.4(1.7-6.2) | 3.93 (1.84 – 6.70) | 1.71 (0.94 – 3.11) | <0.001 |
| eGFR at admission (ml/min) (‡): missing (8) | 20.1 (9.2-48.3) | 15.28 (6.75 – 41.15) | 44.58 (22.11 – 89.41) | <0.001 |
| Peak creatinine (mg/dl) | 5.0 (2.8-7.6) | 5.93 (3.55 – 8.39) | 2.12 (1.52 – 4.05) | <0.001 |
| Fibrosis  <25%  26-50%  51-75%  >76% | 131 (94%)  6 (4%)  1 (1%)  1 (1%) | 94 (93%)  6 (6%)  1 (1%)  0 (0%) | 37 (97%)  0 (0%)  1 (2%) | 0.148 |
| Tubular atrophy  <25%  26-50%  51-75%  >76% | 152 (94%)  6 (4%)  1 (1%)  2 (1%) | 106 (93%)  6 (5%)  1 (1%)  1 (1%) | 46 (98%)  0 (0%)  1 (2%) | 0.335 |
| Glomerulosclerosis | 0 (0 – 0.11) | 0 (0 – 0.18) | 0 (0 – 0.07) | 0.302 |
| Need for Acute KRT (%) | 64 (40) | 58 (51) | 6 (13) | <0.001 |
| Duration on dialysis(days) | 19 (6-29) | 11(7-22) | 20(5-75) | 0.583 |
| Interval between drug withdrawal and kidney Biopsy (days) | 7(5-10) | 7(4-11) | 6(4-9) | 0.036 |
| Interval between diagnosis of AIN and GC (days) |  | 1(0.7-1.1) |  | N/A |
|  |  |  |  |  |

(‡): – eGFR not calculated for 8 patients under 18 years old (CKD-EPI formula not applicable). Values are presented as median (interquartile range) or n (%), unless otherwise specified. N/A not available.

Table S2. Kidney outcomes.

| Variable | Total  n = 161 | Treated with glucocorticoids (GC)  n = 114(71%) | Standard treatment  n = 47(29%) | p |
| --- | --- | --- | --- | --- |
| Creatinine at admission (mg/dl) | 3.4(1.7-6.2) | 3.93 (1.84 – 6.70) | 1.71 (0.94 – 3.11) | <0.001 |
| eGFR at admission (ml/min) (‡): missing (8) | 20.1 (9.2-48.3) | 15.28 (6.75 – 41.15) | 44.58 (22.11 – 89.41) | <0.001 |
| Peak creatinine (mg/dl) | 5.0 (2.8-7.6) | 5.93 (3.55 – 8.39) | 2.12 (1.52 – 4.05) | <0.001 |
| Creatinine at discharge (mg/dl) | 1.69(0.9-2.1) | 1.73 (1.06 – 2.67) | 1.27 (0.90 – 1.91) | 0.003 |
| eGFR at discharge (ml/min) (‡): missing (8) | 48.5 (28.0-65.9) | 42.1 (20.3 – 68.42) | 60.0 (38.45 – 102.3) | <0.001 |
| Delta Creatinine (mg/dl) | -3.0(-5.3 - -0.6) | -3.12 (-6.00 – -1.34) | -0.72 (-2.54 – -0.10) | <0.001 |
| Need for Acute KRT (%) | 64 (40) | 58 (51) | 6 (13) | <0.001 |
| Permanent dialysis (%) | 12 (7.45) | 11 (9.6) | 1 (2.1) | 0.997 |

(‡): – eGFR not calculated for 8 patients under 18 years old (CKD-EPI formula not applicable). Values are presented as median (interquartile range) or n (%), unless otherwise specified. N/A not available.
